# Role of the Early Detection and Prevention of Dental Caries in Children: A Systematic Review of Clinical Outcomes

**DOI:** 10.7759/cureus.85185

**Published:** 2025-06-01

**Authors:** Yashab James, Ayesha Nadeem, Fazal Carpenter

**Affiliations:** 1 Dentistry, Mission Hospital, Peshawar, PAK; 2 Dentistry, University of Poonch, Rawalakot, PAK; 3 Dentistry, University of Health Sciences, Lahore, PAK; 4 Internal Medicine, Nishtar Medical University, Multan, PAK

**Keywords:** children, dental caries, early detection, fissure sealants, fluoride varnish, oral health education, prevention, prisma, silver diamine fluoride, systematic review

## Abstract

Dental caries is one of the most prevalent chronic conditions affecting children worldwide, contributing significantly to pain, school absenteeism, and reduced quality of life. Despite its preventable nature, early childhood caries continues to pose a major public health challenge, particularly in underserved populations. This systematic review aimed to evaluate the clinical outcomes of early detection and preventive interventions in managing dental caries among children. A comprehensive literature search was conducted in accordance with the Preferred Reporting Items for Systematic Reviews and Meta-Analyses (PRISMA) guidelines across PubMed, BioMed Central (BMC) Oral Health, the Journal of Dental Research, and the International Dental Journal. The search covered randomized controlled trials (RCTs) published between January 2023 and January 2024, limited to the English language and pediatric populations. Out of 785 initially identified records, eight RCTs met the eligibility criteria and were included in the final synthesis. The included studies assessed interventions such as fluoride varnish, resin-based and glass ionomer sealants, silver diamine fluoride (SDF), and school-based oral health programs. These strategies were found to significantly reduce caries incidence, arrest lesion progression, and improve oral health behaviors. Fissure sealants and fluoride varnishes demonstrated comparable efficacy, while frequent applications of SDF proved especially effective in arresting early caries. School-based interventions combining education with preventive care emerged as both cost-effective and impactful. Most included studies were of low to moderate risk of bias. This review reaffirms the importance of integrating timely preventive dental strategies into pediatric healthcare and public health systems to curb the burden of caries and promote sustainable oral health outcomes in children.

## Introduction and background

Dental caries is among the most prevalent chronic diseases in children, significantly impacting oral health, overall well-being, and the quality of life [1]. Despite being largely preventable, it continues to affect millions, especially in low- and middle-income countries and underserved populations. Its multifactorial etiology involves dietary patterns, oral hygiene, fluoride exposure, socioeconomic status, and access to dental care [2,3]. If undetected or untreated early, caries can lead to pain, infection, eating and speech difficulties, school absenteeism, and economic burden [4]. Early detection and preventive strategies are crucial in halting lesion progression and preserving dentition. Interventions such as fluoride varnishes, fissure sealants, silver diamine fluoride (SDF), oral health education, and school-based public health initiatives have been evaluated in numerous randomized controlled trials (RCTs), offering insights into their comparative effectiveness [5-7].

This systematic review, developed using a structured patient, intervention, comparison, and outcome (PICO) framework, consolidates findings from rigorously selected randomized controlled trials (RCTs) published between January 2023 and January 2024, with a focus on studies assessed as low to moderate risk of bias using the Cochrane Risk of Bias (RoB) 2.0 tool (London, England) [8]. By restricting inclusion to recent RCTs involving children aged 3-10 years and applying strict methodological criteria, this review prioritizes high-quality evidence defined by proper randomization, clear outcome reporting, and adequate follow-up durations. It evaluates the impact of early detection and prevention strategies for pediatric dental caries, including fluoride applications, SDF treatments, sealants, and school-based programs. In contrast to prior reviews, this synthesis incorporates new data from underserved and underrepresented populations, such as rural schoolchildren in Iraq and Indigenous communities, and explores comparative intervention frequencies, cost-effectiveness outcomes, and programmatic combinations of education with clinical care. Primary outcomes include caries incidence and progression, while secondary outcomes encompass cost-effectiveness and oral health-related quality of life. This review offers an up-to-date, context-sensitive appraisal of early caries prevention approaches, contributing novel insights to both clinical practice and health policy.

## Review

Materials and methods

Search Strategy

The literature search for this systematic review was conducted in accordance with the Preferred Reporting Items for Systematic Reviews and Meta-Analyses (PRISMA) 2020 guidelines to ensure transparency and reproducibility [9]. Searches were carried out in February 2024 across four databases: PubMed, BioMed Central (BMC) Oral Health, the Journal of Dental Research, and the International Dental Journal, focusing on articles published between January 1, 2023, and January 31, 2024. Filters were applied to retrieve only randomized controlled trials (RCTs) published in English and involving human participants within the pediatric age group (3-10 years). The objective was to identify recent evidence on the role of early detection and preventive interventions for dental caries in children. The primary MeSH terms used during the PubMed search included the following: "Dental Caries", "Child", "Fluoride Varnishes", "Dental Sealants", "Silver Diamine Fluoride", and "Prevention and Control".

The initial search yielded 785 records, of which 215 were duplicates. After screening 570 titles and abstracts, 220 full-text articles were retrieved for eligibility assessment. Following the application of predefined inclusion criteria, RCT design, relevance to pediatric preventive dental care, and quantitative reporting of caries-related outcomes, eight studies met the eligibility criteria and were included in the final qualitative synthesis. The inclusion criteria specifically targeted trials evaluating early preventive interventions such as fluoride varnish, fissure sealants, silver diamine fluoride (SDF), and school- or community-based oral health programs, with outcomes including caries incidence, arrest rates, and changes in decayed, missing, and filled teeth (dmft/DMFT) scores.

Eligibility Criteria

This systematic review included only randomized controlled trials (RCTs) published in English within the past year that assessed the impact of early detection and preventive interventions for dental caries in children aged 3-10 years. Eligible studies evaluated strategies such as fluoride varnish, fissure sealants, silver diamine fluoride (SDF), and school- or community-based oral health programs and reported quantitative outcomes such as caries incidence, arrest rates, or DMFT/dmft scores. Studies were excluded if they involved adults, lacked randomization, focused on unrelated topics, or did not provide clear outcomes or follow-up data, ensuring methodological rigor and relevance to the research objective.

Data Extraction

Data extraction was performed systematically from each included study using a predesigned template that captured key information such as study design, population characteristics, sample size, the type of intervention, comparator, the duration of follow-up, outcome measures, and main conclusions. Data were extracted independently to minimize errors and ensure accuracy. Particular attention was given to the type and frequency of preventive interventions, outcome assessment methods (e.g., DMFT/dmft indices, International Caries Detection and Assessment System {ICDAS} scores, and lesion arrest rates), and reported statistical findings. Any discrepancies or unclear data were clarified through consensus discussions or by revisiting the full-text articles. This standardized approach ensured consistency and allowed for effective comparison across studies.

Data Analysis and Synthesis

Data analysis and synthesis were conducted qualitatively due to the heterogeneity in interventions, outcome measures, and follow-up durations among the included studies. A narrative synthesis was used to compare and summarize findings across studies, highlighting patterns, consistencies, and variations in clinical effectiveness and public health impact. Studies were grouped according to intervention type and evaluated for comparative effectiveness, with an emphasis on both short-term and long-term outcomes. Additionally, a risk of bias assessment was performed using the Cochrane RoB 2.0 tool to evaluate the methodological quality of each study, and the findings were integrated into the synthesis to contextualize the strength and reliability of the evidence [10].

Results

Study Selection Process

**Figure 1:**
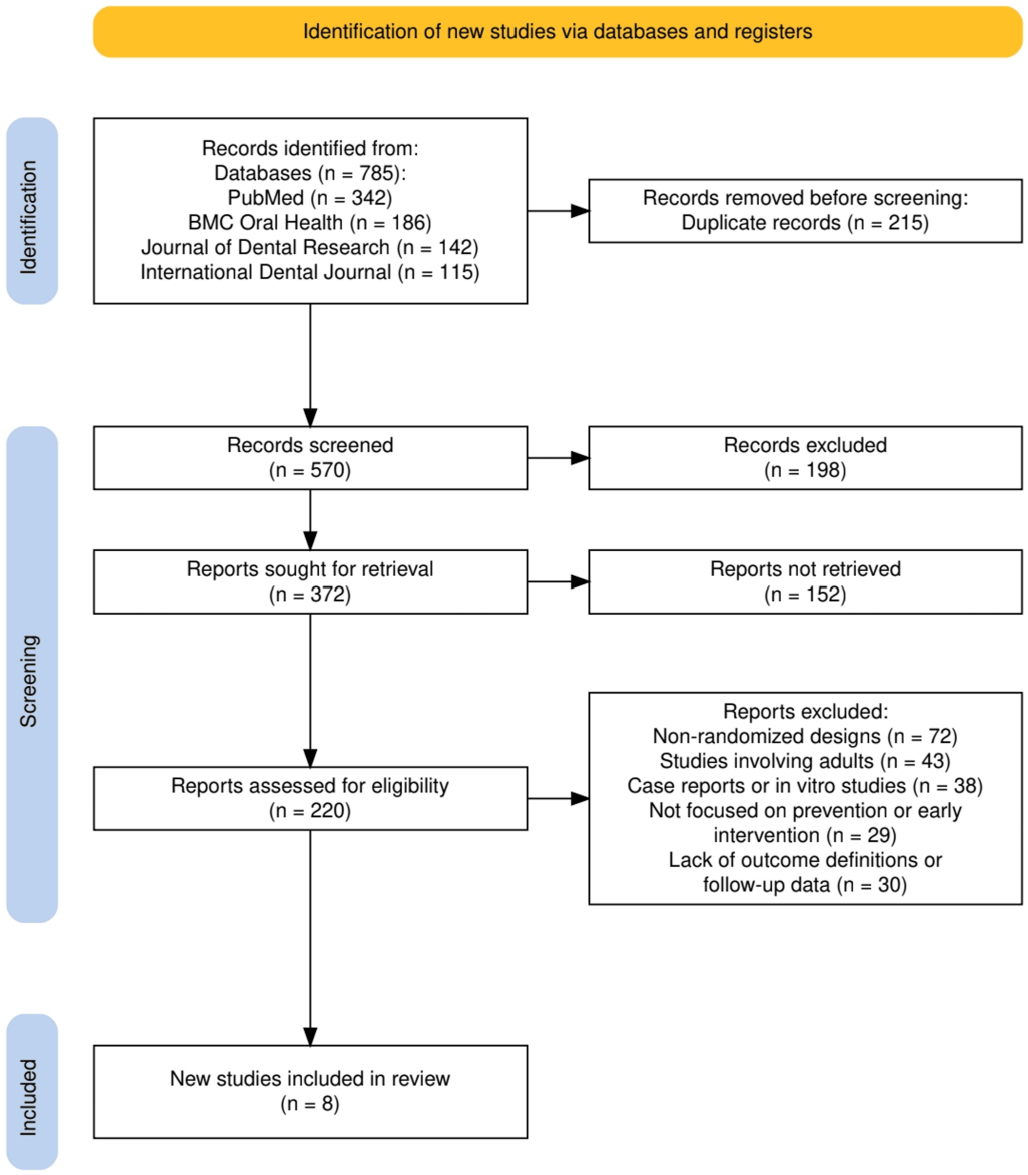
The PRISMA flowchart represents the study selection process. PRISMA, Preferred Reporting Items for Systematic Reviews and Meta-Analyses; BMC, BioMed Central

The study selection process followed the PRISMA 2020 guidelines and is illustrated in Figure 1. A total of 785 records were identified through database searches, including PubMed (342), BMC Oral Health (186), the Journal of Dental Research (142), and the International Dental Journal (115). After the removal of 215 duplicate records, 570 records remained for screening. During the screening phase, 198 records were excluded based on title and abstract review, leaving 372 full-text reports sought for retrieval. Of these, 152 reports could not be retrieved. The remaining 220 reports were assessed for eligibility, out of which 212 were excluded for reasons including non-randomized design (n = 72), adult population (n = 43), case reports or in vitro studies (n = 38), the lack of focus on early intervention or prevention (n = 29), and insufficient outcome or follow-up data (n = 30). Ultimately, eight studies met the inclusion criteria and were included in the qualitative synthesis.

Characteristics of the Selected Studies

The characteristics of the selected studies are summarized in Table 1 and demonstrate notable diversity in design, population, intervention type, and outcome measures. All eight included studies were randomized clinical trials, with designs ranging from double-blind and split-mouth to cluster-randomized and open-label trials. The study populations primarily included children aged 3-10 years, with sample sizes varying from 84 to 1,335 participants, reflecting both clinical and community-based settings. Interventions assessed across the studies included fluoride varnish, glass ionomer sealants, resin-based fissure sealants, and silver diamine fluoride, either individually or in combination, with several studies incorporating oral health education as part of broader prevention strategies. Comparators ranged from alternative fluoride formulations to no-treatment or education-only groups. The follow-up periods spanned from six months to 60 months, and outcome measures included caries incidence, dmft/DMFT scores, lesion arrest rates, and behavioral improvements. Collectively, the included studies provided a comprehensive view of preventive strategies for early childhood caries across diverse populations and intervention models.

**Table 1:** The summary of the selected studies in the systematic review. d2-d3 lesions: carious lesions classified as extending into enamel (d2) or dentin (d3). CPP-ACP, casein phosphopeptide-amorphous calcium phosphate; TCP, tricalcium phosphate; DMFT, decayed, missing, and filled teeth (permanent dentition); dmft, decayed, missing, and filled teeth (primary dentition); ECC, early childhood caries; ICDAS, International Caries Detection and Assessment System; DMFS, decayed, missing, and filled surfaces

Study (First Author and Year)	Study Design	Population Characteristics (Age and Sample Size)	Intervention	Comparator	Duration/Follow-Up	Outcome Measures	Conclusions
Uhlen-Strand et al., 2024 [11]	Pragmatic split-mouth randomized controlled trial (RCT)	Children aged 6-10 years at high caries risk (n = 409)	Resin-based fissure sealants (applied once, maintained as per usual practice)	Fluoride varnish (FV) (applied at baseline, six, and 12 months)	36 months	Success = no need for invasive treatment; failure = dentin lesion or restoration	Fissure sealants were statistically more effective than fluoride varnish, though the difference did not exceed the clinically important threshold of 10%
Manchanda et al., 2024 [12]	Double-blind RCT	Healthy preschool children aged 3-4 years, n = 582 (with at least one carious lesion)	5% sodium Fluoride (NaF) varnish with CPP-ACP (MI Varnish™) or with TCP (Clinpro™ White), applied quarterly	Conventional 5% NaF varnish (Duraphat®), applied quarterly	24 months	Incidence of new caries; increment in cavitated and non-cavitated lesions	No significant difference in caries prevention among the three fluoride varnishes; all showed similar efficacy in high-risk children
Tang et al., 2024 [13]	Randomized controlled trial with cost-effectiveness analysis	Children aged 6-8 years in rural Guangxi, China, n = 1,335	Fluoride varnish application + oral health education every six months	Oral health education alone (no FV)	3 years	Caries prevalence, DMFT index, incremental cost-effectiveness ratio (ICER), cost-benefit ratio (CBR)	Fluoride varnish combined with education was more cost-effective and resulted in fewer caries compared to education alone; recommended for policy implementation in rural areas
Ju et al., 2024 [14]	Outcome assessor-blinded, cross-in randomized controlled trial	Pregnant Aboriginal women and their children, n = 448 (follow-up to child age of 60 months)	Early ECC intervention: maternal dental care during pregnancy; fluoride varnish at six, 12, 18 months; motivational interviewing; and anticipatory guidance	Same intervention delayed until the child was 24 months old	24, 36, and 60 months	Mean dmft scores at three time points; risk ratio for ECC	Early intervention significantly reduced ECC at all follow-up points and showed both immediate and long-term preventive effects among Indigenous children
Lam et al., 2024 [15]	Randomized controlled trial	Preschool children from 18 kindergartens, n = 736	Single application of glass ionomer sealant (GIS) at baseline	Quarterly application of 5% sodium fluoride varnish (NaFV)	18-24 months	Proportion of primary second molars (PSMs) developing dentinal caries (ICDAS ≥ 4)	GIS and NaFV showed similar effectiveness in preventing occlusal caries; choice can depend on clinical or parental preference
Ghasemi et al., 2025 [16]	Cluster randomized controlled trial	Iraqi schoolchildren aged 8-10 years, n = 372	School-based oral health education + single application of 5% sodium fluoride varnish	Oral health education only	6 months (caries outcomes); three months (behavioral outcomes)	Caries increment (DMFS, DMFT, and ICDAS), changes in oral health behaviors	The combined fluoride varnish and education program significantly reduced caries incidence and improved oral hygiene behaviors compared to education alone
Schroth et al., 2024 [17]	Open-label, parallel-group randomized clinical trial	Children with cavitated carious lesions (mean age of ~44.4 months), n = 84	38% silver diamine fluoride (SDF) + 5% NaF varnish applied at one-month or four-month intervals	Same SDF + NaF protocol applied every six months	Varies per group; up to final follow-up after the second visit	Caries arrest rate based on lesion hardness and color	SDF applied at one- or four-month intervals was significantly more effective in arresting ECC than six-month intervals; frequent applications are superior
Padilla Cáceres et al., 2024 [18]	Open-label clinical trial with single group assignment	Preschool children aged 3-4 years with ≥1 active d2-d3 carious lesion, n = 237	Sequential application of 38% SDF gel + 2.5% NaF varnish, repeated at five months	No comparator	12 months	Arrest of d2-d3 carious lesions; parental satisfaction	Over 90% of carious surfaces were arrested after one year; high parental acceptance despite discoloration

Quality Assessment

The quality assessment of the included studies, summarized in Table 2, was conducted using the Cochrane Risk of Bias (RoB) 2.0 tool, which evaluates key domains including the randomization process, deviations from intended interventions, missing outcome data, outcome measurement, and the selection of reported results. Three studies were assessed as having a low overall risk of bias, indicating strong methodological rigor and minimal threats to internal validity. Four studies were categorized as having some concerns, primarily due to open-label designs, unclear reporting of randomization procedures, or cluster allocation methods that were not fully explained. One study was rated as high risk of bias, largely due to its non-randomized, single-group design without a comparator, which limits the ability to draw causal inferences. Overall, the majority of studies demonstrated acceptable methodological quality, supporting the reliability of the evidence synthesized in this review.

**Table 2:** The quality assessment of each of the selected studies.

Study (First Author and Year)	Randomization Process	Deviations From Intended Interventions	Missing Outcome Data	Measurement of the Outcome	Selection of the Reported Result	Overall Risk of Bias
Uhlen-Strand et al., 2024 [11]	Low risk	Low risk	Low risk	Low risk	Low risk	Low risk
Manchanda et al., 2024 [12]	Low risk	Low risk	Low risk	Low risk	Low risk	Low risk
Tang et al., 2024 [13]	Some concerns (secondary analysis)	Low risk	Low risk	Low risk	Some concerns	Some concerns
Ju et al., 2024 [14]	Low risk	Some concerns (timing differences in groups)	Low risk	Low risk	Low risk	Some concerns
Lam et al., 2024 [15]	Low risk	Low risk	Low risk	Low risk	Low risk	Low risk
Ghasemi et al., 2025 [16]	Some concerns (cluster randomization not clearly reported)	Low risk	Low risk	Low risk	Some concerns	Some concerns
Schroth et al., 2024 [17]	Low risk	Some concerns (open-label design)	Low risk	Low risk	Low risk	Some concerns
Padilla Cáceres et al., 2024 [18]	High risk (non-randomized single group)	High risk	Low risk	Low risk	High risk	High risk

Discussion

The systematic review synthesized evidence from eight randomized clinical trials assessing the impact of various early detection and preventive strategies for dental caries in children. The findings consistently demonstrate that both fluoride-based interventions and sealant applications are effective in reducing caries incidence and arresting lesion progression across different age groups and settings. Fissure sealants showed slightly higher clinical effectiveness than fluoride varnish over a 36-month period, while no significant difference was observed between different fluoride varnish formulations in high-risk preschoolers. School-based programs that combined fluoride varnish with oral health education were more effective than education alone and proved cost-effective. Comprehensive early childhood interventions that included maternal dental care and the early application of varnish yielded sustained reductions in caries burden among Indigenous populations. Similarly, silver diamine fluoride (SDF) applications, especially when applied more frequently or in combination with sodium fluoride varnish, were highly effective in arresting severe early childhood caries. Overall, the review highlights the effectiveness of early preventive strategies in mitigating the burden of dental caries, with several interventions showing promising outcomes in diverse pediatric populations.

The findings of this review align with and reinforce existing literature emphasizing the efficacy of early preventive interventions in reducing dental caries among children [19]. Prior studies have consistently shown that fluoride varnish and sealants are effective in caries prevention, with systematic reviews and meta-analyses supporting their widespread use in pediatric populations [20]. The comparable effectiveness observed between different fluoride varnish formulations echoes previous research indicating that the presence of additional calcium-phosphate compounds may not significantly enhance preventive outcomes. Moreover, the superior performance of fissure sealants in high-risk groups corroborates earlier evidence suggesting their benefit in sealing deep pits and fissures prone to caries development [21]. The results from school-based and community-driven programs are consistent with public health models advocating for integrative approaches that combine education with clinical preventive measures. Furthermore, the effectiveness of silver diamine fluoride in arresting carious lesions supports the growing body of literature advocating its use as a minimally invasive, cost-effective solution, especially in populations with limited access to restorative care [22]. Collectively, the review contributes to the existing knowledge by confirming and contextualizing the utility of these interventions across varied settings and risk profiles.

The included studies in this review exhibit several strengths, notably their randomized controlled designs, which enhance internal validity and reduce the risk of selection bias. Most studies had adequate sample sizes, clear outcome definitions, and appropriate follow-up durations, allowing for meaningful comparisons between interventions. Additionally, the inclusion of diverse populations, from high-risk preschoolers to underserved communities, adds to the generalizability of the findings. However, certain limitations were also evident. Some studies, particularly those using open-label or single-group designs, lacked blinding, which could introduce performance or detection bias. In cluster-randomized or community-based trials, variations in implementation fidelity and participant adherence may have influenced outcomes. Furthermore, heterogeneity in intervention protocols, outcome assessment methods, and follow-up periods may complicate direct comparisons across studies. Despite these limitations, the overall methodological quality was acceptable, and the collective evidence provides a robust foundation for evaluating early preventive strategies in pediatric dental care.

This review highlights the vital role of early, evidence-based preventive strategies, such as fluoride varnish, fissure sealants, and silver diamine fluoride, in reducing the global burden of dental caries in children, particularly when tailored to individual risk profiles and delivered through school- or community-based programs [7,23]. These low-cost, practical interventions are especially valuable in underserved populations with limited access to restorative care, reinforcing prevention as a foundation of pediatric oral health. The findings support the integration of such strategies into routine healthcare and education systems, urging policymakers to expand access in high-risk communities and develop standardized guidelines on intervention frequency and combinations to optimize oral health outcomes at the population level [24].

Future research should focus on the long-term comparative effectiveness of various preventive strategies across broader demographic and geographic populations. Studies that explore optimal application frequencies, patient adherence, and cost-effectiveness in real-world settings are needed to strengthen evidence-based guidelines. Additionally, trials incorporating behavioral, educational, and systemic health factors could further clarify the role of multifaceted interventions [25]. There is also a need for high-quality randomized studies assessing newer technologies and formulations, such as novel SDF gels or bioactive sealants, to continue advancing minimally invasive pediatric dental care.

## Conclusions

This systematic review highlights the strong evidence supporting early detection and preventive strategies in reducing the burden of dental caries among children. Interventions such as fluoride varnish, fissure sealants, and silver diamine fluoride, particularly when applied frequently or in combination, are clinically effective across various pediatric populations and settings. School- and community-based programs that integrate education with preventive care offer an especially impactful and scalable approach. The key takeaway is that early, evidence-based interventions not only prevent the onset and progression of caries but also promote long-term oral health in children, making them essential components of both clinical practice and public health policy.
